# Lifestyle and Sarcopenia—Etiology, Prevention, and Treatment

**DOI:** 10.5041/RMMJ.10091

**Published:** 2012-10-31

**Authors:** Oren Rom, Sharon Kaisari, Dror Aizenbud, Abraham Z. Reznick

**Affiliations:** 1 Department of Anatomy and Cell Biology, Rappaport Faculty of Medicine, Technion–Israel Institute of Technology, Haifa, Israel and; 2 Orthodontic and Craniofacial Department, Rambam Health Care Campus, Haifa, Israel

**Keywords:** Alcohol intake, cigarette smoking, exercise, nutrition, physical activity, sarcopenia

## Abstract

The term sarcopenia describes the loss of skeletal muscle mass, strength, and function in old age. As the world population continues to grow older, more attention is given to the phenomena of sarcopenia and the search for strategies of prevention and treatment. The progression of sarcopenia is affected by age-related physiological and systemic changes in the body, including alterations in skeletal muscle tissue, hormonal changes, increased inflammatory activities, and oxidative stress. Sarcopenia progression is also affected by lifestyle factors which are far more controllable. These factors include various aspects of nutrition, physical activity, exercise, alcohol intake, and tobacco use. Raising the public awareness regarding the impact of these factors, as causes of sarcopenia and potential strategies of prevention and treatment, is of great importance. In this review we aim to describe various lifestyle factors that affect the etiology, prevention, and treatment of sarcopenia.

## INTRODUCTION

The term sarcopenia (in Greek, *sarx* for flesh and *penia* for loss), first proposed by Irwin Rosenberg, describes the age-related loss of skeletal muscle mass and strength.^1^ Sarcopenia is a common impaired state of health with a high personal toll and huge financial costs.^2^ However, sarcopenia has no accepted clinical definition and no codes in the International Classification of Diseases 9th Revision (ICD-9).^2^ Therefore, the European Working Group on Sarcopenia in Older People (EWGSOP), assembled in 2009, developed definitions, diagnostic criteria, categories, and stages in sarcopenia.^2^ According to the EWGSOP, sarcopenia is diagnosed by the presence of low muscle mass along with low muscle function (strength or physical performance).^2^ The EWGSOP suggested the following categories to reflect the severity of sarcopenia: *Pre-sarcopenia*, characterized by low muscle mass with no impact on muscle function; *Sarcopenia*, characterized by low muscle mass plus low muscle strength or low physical performance; and *Severe sarcopenia*, characterized by low muscle mass plus low muscle strength and low physical performance.^2^ The EWGSOP also suggested using healthy young adults as reference populations, with cut-off points at two standard deviations below the mean reference value for muscle mass, muscle strength, and physical performance. Recommended measurement techniques include dual energy X-ray absorptiometry (DEXA) scan for muscle mass, isometric hand grip test for muscle strength, and gait speed test for physical performance.^2^

The prevalence of sarcopenia among people older than 65 years has been estimated as high as 15%, and 50% among people over the age of 80.^3^ As a major public health problem, the health care cost of sarcopenia in the United States alone was estimated at 18.5 billion dollars in the year of 2000.^3^,^4^ This estimation took into consideration the direct costs of sarcopenia, including hospital, out-patient, and home health care expenditures, and did not include the indirect costs of sarcopenia such as loss of productivity.^4^ The world’s population over the age of 60 is expected to triple from 600 million in 2000 to more than 2 billion by the year of 2050.^5^ Owing to this worldwide increase in life expectancy, the prevalence and cost of sarcopenia are likely to rise. Therefore, developing strategies to prevent and treat sarcopenia are of great importance.

From the third decade of life a shift in body composition occurs. Between the ages of 30 and 60, the average adult is expected to gain approximately 0.45 kg (1 lb) of fat and lose about 0.23 kg (0.5 lb) of muscle yearly.^6^ From the age of 60, loss of muscle mass is accelerated and is estimated at 2% annually. Also, decline of muscle strength over the age of 60 is estimated at 3% yearly. The result of these losses is a decrease in total muscle cross-sectional area of about 40% between 20 and 60 years of age.^6^ Loss of muscle mass accompanied by increase in fat mass may lead to a body composition phenotype known as sarcopenic obesity. It was estimated that approximately 30% of men and 10% of women over the age of 80 have sarcopenic obesity.^6^ In addition, aging is associated with alterations in skeletal muscle tissue and low muscle quality. For instance, skeletal muscle is infiltrated by fat and connective tissue, the number and size of muscle fibers are decreased, there is a decrease in motor units, disarrangements of myofilaments, accumulation of reactive oxidative species, and reduction in satellite cell activity and number.^7^

**Figure 1:**
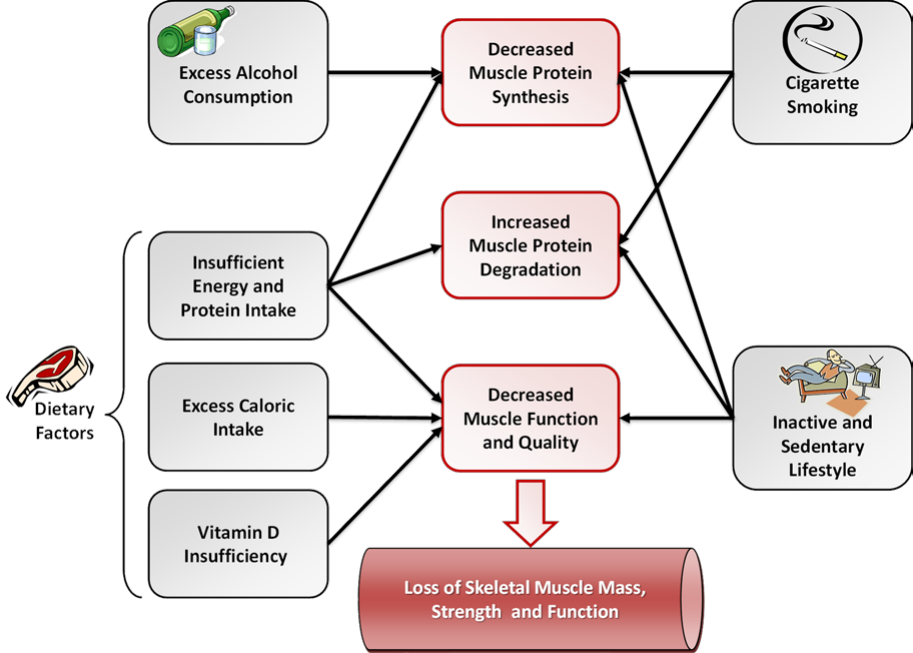
**Lifestyle factors affecting sarcopenia.**

In order to develop strategies to prevent and treat sarcopenia, the risk factors and causes of sarcopenia must be identified. The progression of sarcopenia is affected by age-related systemic changes and by lifestyle habits.^8^ Age-related changes include reduction in anabolic hormones such as testosterone, estrogen, growth hormone, and insulin-like growth factor-1 (IGF-1), increased inflammatory activity, and oxidative stress which contribute to muscle catabolism.^7^ Lifestyle habits have a major impact on sarcopenia as well. These factors include impaired nutrition, reduced physical activity, alcohol consumption, and cigarette smoking.^7^–^9^ A scheme of the effects of these lifestyle factors on skeletal muscle and the progression of sarcopenia is presented (Figure 1). Genetic factors may also affect the progression of sarcopenia. Muscle mass and strength are multifactorial traits that vary widely among individuals. The genetic component of sarcopenia is complex and driven by many genes. Several genes have been identified that contribute to variation of skeletal muscle mass and strength, including the IGF-1 and vitamin D receptor genes.^10^ Since lifestyle factors are more controllable in comparison with age-related systemic changes and genetic factors, it is of great importance to raise the public awareness regarding their influence on the progression of sarcopenia. This review aims to present the importance of lifestyle factors as causes of sarcopenia and potential strategies for prevention and treatment of sarcopenia.

## DIETARY FACTORS IN SARCOPENIA

Aging is associated with reduced appetite and low food intake, which was previously termed the “anorexia of ageing.”^11^ Several causes have been suggested to explain this phenomenon. Anorexia of aging may be the result of early satiety owing to decreased relaxation of the fundus, increased release of cholecystokinin, and increased leptin levels.^6^,^11^ Altered taste and smell, social changes, and economic limitations may also lead to decreased food intake.^12^ These may result in low nutrient intake, which is an important risk factor in the development of sarcopenia. In particular, protein intake has a major influence on skeletal muscle metabolism. Inadequate protein intake is one of the major mechanisms underlying sarcopenia.

The current recommended dietary allowance (RDA) of protein is 0.8 g/kg/day.^3^ It has been estimated that approximately 40% of people over the age of 70 do not meet this RDA.^3^ Furthermore, nitrogen balance studies in aging populations have indicated greater protein needs for the elderly (1.14 g/kg/day) relative to the young (0.8 g/kg/day).^13^ Thalacker-Mercer et al.^14^ assessed the effect of 1 week of inadequate protein intake (0.5 g/kg/day) compared with adequate protein intake (1.2 g/kg/day) on gene expression profiles in skeletal muscle of older adults. It was shown that inadequate protein intake is associated with down-regulation of transcripts associated with protein synthesis, myosin formation, and proliferation of satellite cells.

Increased protein needs in the elderly might be explained by the phenomenon of “anabolic resistance,” a blunted response of muscle protein synthesis following ingestion of dietary protein in the elderly relative to the young.^13^,^15^ This phenomenon is associated with reduction in IGF-1 levels in old age. IGF-1 activates the mammalian target of rapamycin (mTOR) which in turn regulates muscle protein synthesis by initiating translation. Thus, impairment in mTOR signaling leads to decreased capacity and efficiency of protein synthesis.^11^ Previous studies have shown that the elderly are less able efficiently to utilize amino acids for muscle protein synthesis. For instance, Katsanos et al.^16^ examined the effect of essential amino acid (EAA) small bolus (6.7 g) on synthesis of muscle proteins in the elderly compared with the young. It was found that protein synthetic response was diminished in the elderly relative to the young.^13^,^16^ However, Symons et al.^17^ examined muscle protein synthesis in elderly compared with young subjects following ingestion of a 113-g serving of lean beef (approximately 30 g of amino acids). They have shown that muscle synthesis rate was increased equally in both the elderly and the young and concluded that aging does not impair the ability to synthesize muscle protein after ingestion of protein-rich food. These studies demonstrate the importance of the amount of protein ingested and its source in order to stimulate synthesis of muscle protein despite the observed anabolic resistance in the elderly. Also, the timing of protein intake by older adults may be critical to maintain muscle mass. It was suggested that sufficient protein with each meal should be encouraged more than an overall increase in daily protein intake.^12^ Nevertheless, optimal protein intake as a strategy to prevent and treat sarcopenia needs to be further investigated in future studies.

The EAA leucine plays an important role in regulating muscle metabolism and is known as an anti-atrophic agent. Leucine regulates translational control of protein synthesis through activation of the mTOR signaling pathway.^15^ Also, *in-vivo* and *in-vitro* studies have demonstrated the ability of leucine to attenuate skeletal muscle wasting by interaction with proteolytic pathways.^18^ Katsanos et al.^19^ have shown that increasing the proportion of leucine in a mixture of EAA given to elderly subjects can reverse the attenuated response of muscle protein synthesis. Flakoll et al.^20^ have found that 12 weeks of daily supplementation of leucine metabolite β-hydroxy-β-methylbutyrate (HMB) together with arginine and lysine can positively alter measurements of functionality, strength, fat-free mass, and protein synthesis in elderly women. Leucine supplementation to immobilized rats has been shown to reduce muscle wasting via minimizing gene expression of the muscle-specific E3 ligases, muscle ring finger 1 (MuRF1) and muscle atrophy F-box (MAFbx/atrogin-1) of the ubiquitin–proteasome system.^21^ These E3 ligases mediate the ubiquitination of muscle proteins and play an important role in myofibrillar protein breakdown. Knock-out mice lacking these E3 ligases are protected from muscle atrophy.^21^ C2C12 muscle cells treated with 5 mM leucine have demonstrated suppressed MAFbx/atrogin-1 and MuRF1 mRNA levels.^22^ Therefore, leucine supplementation to older adults may serve as a potential strategy to combat the progression of sarcopenia. The dose–response of leucine supplementation is unknown, and future studies should focus on finding effective and safe doses for the use of leucine as an anti-atrophic agent in sarcopenia.^18^ In the meantime, older adults should be encouraged to consume a diet high in EAAs, in particular leucine-rich food sources such as beef, fish, and legumes.^6^

Vitamin D has recently received recognition as another potential intervention modality for sarcopenia.^6^ Recent findings have demonstrated that vitamin D plays an important role in skeletal muscle tissue by maintaining the function of type II fibers, preserving muscle strength and preventing falls.^23^ Vitamin D receptor knock-out mice are characterized by growth retardation, muscle impairment, and smaller diameters of muscle fiber than those of wild-type mice.^24^ Older adults are at increased risk of vitamin D insufficiency due to various factors. As people age, the skin’s ability to synthesize vitamin D efficiently is reduced, and the kidney is less able to convert vitamin D to its active form; in addition, inadequate sunlight exposure which is essential for vitamin D synthesis and low consumption of dietary vitamin D are common among the elderly.^24^–^26^ Indeed, the prevalence of vitamin D insufficiency in the elderly has been estimated at 78%.^26^ Clinical, *in-vivo*, and *in-vitro* studies have shown that vitamin D affects muscle strength and function.^26^ For instance, Bischoff-Ferrari et al.^27^ have shown that higher concentrations of vitamin D are associated with better musculoskeletal function in the lower extremities than lower vitamin D concentrations in people over the age of 60. Also, Pfeifer et al.^28^ have demonstrated that combined vitamin D (800 IU/day) and calcium (1,000 mg/day) supplementation are superior to calcium alone in reducing the number of falls and improving muscle function and strength in community-dwelling older individuals. On the cellular level, *in-vitro* studies have demonstrated that vitamin D can stimulate proliferation and differentiation of myoblasts. Signaling pathways involved in vitamin D-associated proliferation and differentiation of myoblasts include the mitogen-activated protein kinases (MAPK) pathways such as the extracellular signal-regulated kinase 1 and 2 (ERK1/2), p38 MAPK, and c-Jun NH2-terminal 1 and 2 MAPK (JNK1/2).^26^ Nevertheless, the exact mechanisms of vitamin D action in skeletal muscle and how it promotes improvements in muscular performance are yet to be clear, and further studies are needed. The role of vitamin D in skeletal muscle and its ability to prevent muscular deterioration has been demonstrated at all research levels. Supplementation of vitamin D on the basis of measured low levels or in groups at high risk for deficiency appears as an important strategy for the prevention and treatment of sarcopenia.^6^

Another main issue in the context of sarcopenia is weight management and body mass index (BMI). As more attention is given to the prevention of obesity, older people already at risk of sarcopenia may attempt to lose weight when weight stability is more important to them. Older people with BMI within what is considered an ideal range for younger individuals may be at nutritional risk and at risk of sarcopenia.^7^ Weight loss attempts in older adults may lead to caloric insufficiency that can accelerate the progression of sarcopenia.^12^ On the other hand, the issue of sarcopenic obesity must also be taken into consideration. Excess caloric intake that results in obesity may also accelerate sarcopenia.^12^ Obesity is a BMI equal or greater than 30 kg/m^2^.^29^ Sarcopenic obesity is an alternate model of obesity characterized by loss of muscle mass and increased fat mass.^30^ Obese older adults have higher muscle mass than non-obese; however, it was suggested that muscle quality in obese individuals is poor due to increased intramuscular adipose tissue, leading to muscle weakness, frailty, and disability.^12^ In sarcopenic obese individuals, weight loss may be necessary but should be achieved in a way that preserves lean tissue.^7^ This may be achievable through inclusion of an exercise program focusing on resistance training which will be discussed later. Also, during calorie-restricted diets increased protein intake is important to maintain muscle mass especially in sarcopenic obesity.^30^

## PHYSICAL ACTIVITY, SEDENTARY LIFESTYLE, AND SARCOPENIA

Physical activity is defined as any movement produced by the contraction of skeletal muscles that increases energy expenditure. Physical activity includes daily activities such as standing up from a chair and climbing stairs, as well as intentional movements for health benefits such as walking or biking.^31^ Persons performing only baseline physical activities such as standing, walking slowly, and lifting light objects are considered inactive. Physical activities added to these baseline activities produce substantial health benefits.^31^ Exercise is planned, structured, and repetitive physical activity performed during leisure time for the purpose of maintaining or improving the components of physical fitness, functioning, and health.^31^ Older adults who are less physically active are more likely to have lower skeletal muscle mass and strength and are at increased risk of developing sarcopenia.^3^

Sedentary behavior refers to activities that do not substantially increase energy expenditure above the resting level. It includes sleeping, sitting, lying down, and watching television.^32^ Sedentary lifestyle has been shown to be a major risk factor for chronic disease, frailty, and sarcopenia as well.^33^ Studies dealing with the effects of bed rest on skeletal muscle demonstrate the impact of sedentary behavior on muscle mass and metabolism. Only 7 days of recumbency has been shown to result in rapid loss of muscle mass. More prolonged periods of bed rest have resulted in 30% reduction of muscle volume, particularly in muscles of the lower limbs.^34^ Studies examining the effect of immobilization on skeletal muscle have shown a disruption in the balance between protein synthesis and breakdown in which muscle protein anabolism is reduced and catabolism is increased.^34^ Studies conducted on immobilized animals have demonstrated that the damage caused to skeletal muscle is associated with activation of various proteolytic systems which are further activated in muscles of old animals in comparison with young animals.^35^ For instance, increased ubiquitination of myosin heavy chain (MyHC) protein was observed in muscles of old immobilized animals in comparison with young immobilized animals.^35^ Bar-Shai et al.^36^,^37^ have suggested that activation of extracellular hydrolytic and proteolytic systems differ in muscles of old animals compared to young animals during immobilization. A different activation pattern of nuclear factor kB (NF-kB) in muscle atrophy was observed in which the canonic activation pathway of NF-kB was more prominent in muscles from old animals compared to young ones. Also, the involvement of growth hormone in muscular damage and atrophy during limb immobilization was demonstrated by Carmeli et al.^38^ It was shown that administration of growth hormone to old rats significantly reduced muscle weight loss and atrophy, protein oxidation, and fiber disorientation caused by immobilization.

Since low physical activity and sedentary lifestyle are main causes of sarcopenia, exercise is a primary strategy in the prevention and treatment of sarcopenia. Both aerobic training and resistance training can improve the rate of decline in muscle mass and strength with age.^3^ Aerobic training, in which large groups of muscle move for a prolonged period of time, is less likely to contribute to muscle hypertrophy; however, it can increase the cross-sectional area of muscle fibers, mitochondrial volume, and enzyme activity. Also, aerobic exercise can reduce intramuscular fat and improve muscle functionality.^3^ Interestingly, several studies have demonstrated the anabolic effects of aerobic training. Robinson et al.^39^ have shown that 6 weeks of aerobic training in older adults resulted in increased long-term synthesis of muscle protein and DNA in comparison with young sedentary subjects. Pasini et al.^40^ have examined the effect of aerobic treadmill exercise on muscle anabolic pathways in young versus old rats. They have found that aerobic training ameliorated aging-associated impairments in muscle anabolic pathways, affecting the insulin and mTOR signaling pathways.^38^ In addition, Timmerman et al.^41^ have reported that aerobic training in older adults improves nutrient delivery to muscle, thus inducing an increased anabolic effect of nutrient intake.

In comparison to aerobic training, resistance training has a greater effect on increasing muscle mass and strength and attenuates the development of sarcopenia.^3^ Resistance training is a form of exercise in which muscle contracts against an external load. Equipment commonly used to perform resistance training includes free weights, exercise machines, body weight, and elastic bands.^42^ Resistance training increases muscle mass through direct stimulation of muscle protein synthesis already after a few hours of an acute bout of exercise.^43^ The molecular mechanism of resistance training in which synthesis of muscle protein is increased includes the MAPK and mTOR signaling pathways. Following resistance training exercise, phosphorylation of ERK1/2 MAPK is increased and mTOR is activated, leading to activation of downstream translation initiation factors and thus resulting in increased muscle protein synthesis.^43^ Numerous studies have demonstrated the effectiveness of resistance training in improving muscle mass and strength in the elderly. For instance, Frontera et al.^44^ have shown that a 12-week strength training program of 3 days a week in older adults resulted in increased muscle strength, muscle hypertrophy, and myofibrillar protein turnover. Moreover, improvements in muscle strength in older adults have been shown to be achieved with as little as one resistance training session per week.^3^ Taaffe et al.^45^ have shown that a resistance training program of only 1 day per week in older adults improves muscle strength in a similar manner to a resistance training program of 3 days per week.

Progressive resistance training (PRT), in which the load is systematically increased as the person is able to work against a heavier load, is the most commonly used resistance therapy in older people.^3^,^42^ It has been shown to produce large increases in muscle strength, physical function, and lean body mass.^3^,^42^ According to the guidelines for physical activity in older adults by the American College of Sports Medicine and American Heart Association,^46^ in order to maintain or increase muscular strength and endurance, resistance training sessions at a minimum of two non-consecutive days per week should be performed. A progressive weight training program is recommended to include 8–10 exercises for the major muscle groups using a resistance that allows 10–15 repetitions for each exercise.^46^ Mayer et al.^47^ recommended that PRT programs aiming to reduce sarcopenia should consist of three training units per week. Exercises should include 8–12 repetitions per muscle group in 60%–80% of the one-repetition maximum.

Healthy aging adults should be entirely capable of safe participation in PRT programs.^48^ Moreover, resistance training appears to be safe to perform even in participants with multiple co-morbidities.^3^ However, among aged individuals with existing morbidities, careful risk stratification is necessary to ensure safety during resistance training.^48^ Familiarization to the resistance training program, including a period of low-intensity training, is important for novice trainees, especially for the elderly. Following this familiarization period older adults may benefit from a more gradual increase in training intensity to accommodate improvements in strength and muscle hypertrophy.^48^

To summarize, an inactive and sedentary lifestyle is the main factor in the loss of muscle mass and strength of old age. Exercise programs focusing on PRT combined with aerobic training are of great importance in the prevention and treatment of sarcopenia.

## INTERACTIONS BETWEEN NUTRITION AND EXERCISE

Although PRT is a promising strategy for countering sarcopenia, the cellular anabolic response to resistance training is blunted in older adults compared to the young.^13^ This may be the result of greater susceptibility to load-induced myofiber damage, attenuated regenerative capacity, and limited myofiber plasticity in response to resistance training in the elderly.^48^ Adequate dietary intake may promote muscle anabolism and overcome the blunted cellular response in older adults participating in various exercise programs, particularly resistance training.

First, adequate energy intake in elderly during resistance training program is extremely important. Singh et al.^49^ have demonstrated that increased caloric intake can improve muscle strength and growth in elderly who consumed less than the RDA for energy intake. They found that older adults participating in resistance training and taking a 360 calories nutritional supplement increased their muscle strength and type II muscle fiber area significantly when compared with older adults taking part in resistance training alone.

Second, increased protein intake may improve the anabolic response to resistance training in the elderly. It appears that EAAs and in particular leucine play the predominant role in promoting a positive muscle protein balance.^50^ Kim et al.^51^ have examined the effect of exercise with or without supplementation of a leucine-rich EAA mixture on muscle mass and strength in 155 elderly sarcopenic women. They have found that the greatest increase in muscle mass and strength was in the exercise plus EAA supplementation group. Vukovich et al.^52^ have investigated whether the leucine metabolite HMB, administered at a dose of 3 g a day, would benefit 70-year-old adults undergoing a resistance training program in a randomized control study. Compared with the placebo group, the HMB-supplemented group presented increased gain of fat-free mass and loss of body fat. Older adults who are reluctant to use nutritional supplementation may benefit from the consumption of EAAs from food products. Milk-based proteins are an effective protein source for stimulating synthesis of muscle protein and promoting gains in muscle mass.^50^ Bovine milk contains a relatively high proportion of leucine. Also, milk contains both whey and casein proteins, which have different absorption rates. Whey protein has been hypothesized to promote rapid muscle protein synthesis, while casein promotes sustained synthesis of muscle protein.^50^ The timing of EAA-rich protein consumption relative to the resistance training bout may also play an important role in the anabolic response. Resistance training induces increased blood-flow and utilization of amino acids for muscle protein synthesis. Therefore, milk-based proteins should be consumed in close proximity to the resistance training session.^50^ Also, the elderly, in comparison to the young, may require a greater amount of protein to achieve an anabolic response to resistance training. Yang et al.^53^ have reported that muscle protein synthesis in older adults is increased with ingestion of 40 g of whey protein, whereas in younger adults post-exercise rates of muscle protein synthesis are saturated with only 20 g of protein.

The creatine/phospho-creatine energy system is used to sustain adenosine triphosphate (ATP) levels during times of high energy demand as in resistance training bouts.^54^ Previous studies have reported an age-associated reduction in skeletal muscle creatine/phospho-creatine.^54^ Rawson et al.^54^ reviewed the effect of creatine supplementation on skeletal muscle of the elderly. They have reported that supplementation of creatine in older adults, in combination with resistance training, increases lean body mass, enhances fatigue resistance, increases muscle strength, and improves performance of activities of daily living to a greater extent than resistance training alone. Although reported to be a safe dietary supplement, the safety of creatine supplementation and its long-term benefits to the elderly population need to be further investigated before including it as a recommended strategy for the prevention and treatment of sarcopenia.^54^

In summary, to maximize the benefits of exercise in older adults as a method to combat sarcopenia progression, adequate dietary intake is of great importance. This includes sufficient caloric intake and consumption of EAA-rich protein sources that would promote muscle anabolism, especially in older persons taking part in resistance training programs.

## ALCOHOL CONSUMPTION AND SKELETAL MUSCLE

Alcohol misusers frequently suffer from low muscle mass and strength, muscle pain, cramps, difficulties in gait, and falls.^55^ This phenomenon is known as alcoholic myopathy.^55^ Acute alcoholic myopathy occurs after severe alcoholic binges in malnourished alcoholics. It is a rare condition characterized by painful muscles, myoglobinuria, raised serum creatine kinase activities, and often renal impairment.^55^ However, chronic alcoholic myopathy is a common complication of alcoholism affecting approximately 50% of alcohol misusers.^55^ Chronic alcoholic myopathy is not associated with nutritional, vitamin, or mineral deficiencies or alcoholic liver disease, and it is reversible within 6–12 months of abstinence.^55^ Chronic alcoholic myopathy is characterized by selective atrophy of type II muscle fibers, leading to reduction of muscle mass by up to 30%.^55^

Previous studies attempted to explain the molecular mechanisms of alcohol-induced skeletal muscle damage. Tiernan and Ward^56^ administered ethanol acutely to rats and investigated its effects on whole-body and muscle protein synthesis. They have found that ethanol decreased whole-body and muscle protein synthesis by 41% and 75%, respectively. Reilly et al.^57^ studied the effects of ethanol on skeletal muscle protein synthesis and protease activities in rats. Compared with pair-fed controls, significant reductions in skeletal muscle protein, RNA, and DNA contents were found after 24 hours of ethanol administration. Fractional rate of muscle protein synthesis was reduced, though protease activities were not significantly affected by ethanol, indicating that alcohol-induced muscle damage is associated with impaired synthesis of muscle protein and is not promoted by increased activation of proteolytic systems.^55^ Lang et al.^58^ have shown that rats on a 14-week alcohol-containing diet presented an alcoholic myopathy phenotype confirmed by reduced skeletal muscle mass. Their findings also indicated that chronic alcohol consumption impairs translation initiation in muscle by altering activities of several eukaryotic initiation factors. Later, Lang et al.^59^ have shown that acute intraperitoneal administration of alcohol impairs the IGF-1 signaling pathway in skeletal muscle of rats, a key regulator of muscle anabolism. Vary et al.^60^ reported that acute intraperitoneal and oral administration of alcohol increased the expression of muscle-specific E3 ligases MuRF1 and MAFbx/atrogin-1 in skeletal muscles of rats. However, this up-regulation was not associated with increased long-term rates of muscle proteolysis. Therefore, it has been concluded that the loss of muscle mass in response to chronic alcohol abuse results primarily from reduced synthesis of muscle proteins and not increased degradation.^60^

Alcohol abuse appears to affect skeletal muscle severely, promoting its damage and wasting. The above *in-vivo* studies indicate that alcohol-induced muscle damage may be the result of impaired synthesis of muscle protein rather than increased muscle catabolism. Although alcohol consumption is not known as a direct cause of sarcopenia, studies demonstrating the adverse effects of alcohol on skeletal muscle suggest that chronic alcohol consumption may promote loss of muscle mass and strength in old age. Therefore, it is proposed that high alcohol intake is a lifestyle habit that may promote sarcopenia. Reducing alcohol consumption may serve as a strategy for the prevention of sarcopenia.

## CIGARETTE SMOKING AND SARCOPENIA

Cigarette smoking is associated with poor lifestyle habits, such as low levels of physical activity and impaired nutrition.^8^ However, smoking itself is another lifestyle habit that has been found to be associated with sarcopenia in previous studies.^8^ Castillo et al.^61^ examined sarcopenia risk factors in 1,700 community-dwelling men and women aged 55–98 years. They have found that men and women who were current smokers were more likely to have sarcopenia. Szulc et al.^62^ investigated risk factors for sarcopenia in a large cohort of 845 men aged 45–85 years. They have reported that smokers had lower relative appendicular skeletal muscle mass than did subjects who never smoked and that men with sarcopenia smoked significantly more. In addition, Lee et al.^63^ studied the association between sarcopenia and lifestyle factors in 4,000 community-dwelling Chinese elderly over 65 years of age. Similarly, they have found that cigarette smoking is associated with low appendicular skeletal muscle mass. All of the above studies concluded that tobacco smoking is a risk factor for sarcopenia.^61^–^63^

Several studies attempted to explain the mechanism by which cigarette smoking promotes muscle catabolism and accelerates the progression of sarcopenia. The effects of cigarette smoking on skeletal muscle structure and metabolism were demonstrated in clinical, *in-vivo*, and *in-vitro* studies. Montes de Oca et al.^64^ explored the effects of smoking on skeletal muscle by studying biopsies of the vastus lateralis muscle from smokers and healthy control subjects. They have found structural and metabolic damage in skeletal muscle of smokers, including decreased cross-sectional area of type I muscle fibers, and a similar trend in type IIa fibers of smokers. Petersen et al.^65^ studied the effect of smoking on protein metabolism in skeletal muscle of smokers and non-smokers about the age of 60. They have found that the fractional synthesis rate of muscle was significantly lower in smokers compared with non-smokers. Also, smokers presented greater expression of the muscle-specific E3 ligase MAFbx/atrogin-1 and the muscle growth inhibitor myostatin. Therefore, Petersen et al.^65^ concluded that smoking may increase the risk of sarcopenia by impairing muscle protein synthesis and up-regulating genes associated with impaired muscle maintenance. Chronic exposure of animals to cigarette smoke also resulted in muscular damage.^66^–^68^ Mice exposed to cigarette smoke daily for 16 weeks presented a reduction in body and gastrocnemius muscle mass and up-regulation of MAFbx/atrogin-1 and MuRF1 in sampled skeletal muscles.^66^ In addition, 6 months of cigarette smoke exposure to mice resulted in a 20% reduction of force at high-stimulation frequencies.^67^ Barreiro et al.^68^ have also demonstrated that 6 months of cigarette smoke exposure to mice led to reduction in body weight gain and increased oxidative stress in gastrocnemius muscle. In an attempt to understand better the molecular mechanism of cigarette smoke-induced muscle catabolism, we have studied the effects of cigarette smoke exposure on C2 myotubes from an *in-vitro* skeletal muscle cell line. We have found that exposure of C2 myotubes to cigarette smoke caused a decrease in diameter of myotubes, degradation of the main contractile proteins, MyHC and actin, and up-regulation of MAFbx/atrogin-1 and MuRF1. These catabolic processes were mediated by increased intracellular oxidative stress and activation of p38 MAPK. Pretreatment with the antioxidant N-acetyl-cystein (NAC) and inhibition of p38 MAPK prevented cigarette smoke-induced catabolism in C2 myotubes. Based on the above studies and our recent findings, we have suggested a cellular model of cigarette smoke-induced skeletal muscle catabolism.^9^ In this model, components of cigarette smoke may reach skeletal muscle of smokers, leading to increased oxidative stress and activation of signaling pathways which trigger up-regulation of muscle-specific E3 ubiquitin ligases. As a result, degradation of skeletal muscle protein is increased and the progression of sarcopenia in elderly smokers may be accelerated.^9^

## CONCLUSION

Lifestyle habits regarding nutrition, physical activity, exercise, alcohol consumption, and tobacco use have a substantial impact on the progression of sarcopenia and the ability to prevent and treat the loss of muscle mass and function in old age. As life expectancy is increasing worldwide, the prevalence and costs of sarcopenia are expected to rise. In order to treat and delay sarcopenia, the choices we make in our lifestyle habits must be taken into consideration. In contrast to physiological and systemic changes that occur in our body as we age and accelerate the progression of sarcopenia, lifestyle factors are far more controllable. Therefore, raising the public awareness regarding the importance of lifestyle habits on the status of skeletal muscle in old age is of great importance in the management of sarcopenia.

## Abbreviations:

BMI: body mass index;
DEXA: dual energy X-ray absorptiometry;
EAA: essential amino acid;
ERK1/2: extracellular signal-regulated kinase 1 and 2;
EWGSOP: European Working Group on Sarcopenia in Older People;
HMB: β-hydroxy-β-methylbutyrate;
IGF-1: insulin-like growth factor-1;
MAFbx/atrogin-1: muscle atrophy F-box;
MAPK: mitogen-activated protein kinases;
mTOR: mammalian target of rapamycin;
MuRF1: muscle ring finger 1;
MyHC: myosin heavy chain;
PRT: progressive resistance training;
RDA: recommended dietary allowance.
